# Protecting children from unhealthy food marketing: a comparative policy analysis in Australia, Fiji and Thailand

**DOI:** 10.1093/heapro/daad136

**Published:** 2023-11-27

**Authors:** Anne Marie Thow, Sirinya Phulkerd, Yandisa Ngqangashe, Amerita Ravuvu, Liza Zaruba, Carmen Huckel Schneider, Jeff Collin, Ashley Schram, Sharon Friel

**Affiliations:** Faculty of Medicine and Health, Sydney School of Public Health, Menzies Centre for Health Policy and Economics, Charles Perkins Centre (D17), The University of Sydney, Sydney, NSW, 2006, Australia; Institute for Population and Social Research, Mahidol University, Phutthamonthon, Nakhon Pathom, Thailand; Australian Research Centre for Health Equity, School of Regulation and Global Governance, Fellows Road, Australian National University, Canberra, ACT, 2600, Australia; Non-Communicable Disease (NCD) Prevention and Control Programme, Public Health Division, Pacific Community (SPC), Private Mail Bag, Suva, Fiji; Independent Researcher, USA; Faculty of Medicine and Health, Sydney School of Public Health, Menzies Centre for Health Policy and Economics, Charles Perkins Centre (D17), The University of Sydney, Sydney, NSW, 2006, Australia; Global Health Policy Unit, Social Policy, School of Social and Political Science, University of Edinburgh, Edinburgh, EH8 9YL, UK; Australian Research Centre for Health Equity, School of Regulation and Global Governance, Fellows Road, Australian National University, Canberra, ACT, 2600, Australia; Australian Research Centre for Health Equity, School of Regulation and Global Governance, Fellows Road, Australian National University, Canberra, ACT, 2600, Australia

**Keywords:** nutrition, policy, children, marketing, coalition

## Abstract

Restrictions on marketing of unhealthy foods and beverages to children is a globally recommended policy measure to improve diets and health. The aim of the analysis was to identify opportunities to enable policy learning and shift beliefs of relevant actors, to engender policy progress on restrictions on marketing of unhealthy foods to children. We drew on the Advocacy Coalition Framework to thematically analyse data from qualitative policy interviews conducted Australia (*n* = 24), Fiji (*n* = 10) and Thailand (*n* = 20). In all three countries two clear and opposing advocacy coalitions were evident within the policy subsystem related to regulation of unhealthy food marketing, which we termed the ‘strengthen regulation’ and ‘minimal/self regulation’ coalitions. Contributors to policy stasis on this issue were identified as tensions between public health and economic objectives of government, and limited formal and informal spaces for productive dialogue. The analysis also identified opportunities for policy learning that could enable policy progress on restrictions on marketing of unhealthy foods to children as: taking an incremental approach to policy change, defining permitted (rather than restricted) foods, investing in new public health expertise related to emerging marketing approaches and scaling up of monitoring of impacts. The insights from this study are likely to be relevant to many countries seeking to strengthen regulation of marketing to children, in response to recent global recommendations.

**Contribution to Health Promotion**

- This action-oriented research identified lessons to strengthen restrictions on marketing of unhealthy foods and beverages to children
- Strategies include policy design and process factors, such as aiming for incremental changes and shifting to a positive lens
- Building capacity in public health, including expertise in digital marketing, and improving monitoring and reporting are critical

## INTRODUCTION

Marketing of unhealthy foods and beverages contributes to unhealthy diets among children, including through habit formation that persists into adulthood (Boyland *et al.*, 2022a; World Health Organization, 2022). Marketing and advertising act through multiple channels to influence children’s attitudes, preferences and consumption patterns, often towards increased consumption of unhealthy foods and beverages, and is associated with increased body weight (Powell *et al.*, 2017; Smith *et al.*, 2019).

To combat this influence, the World Health Organization (WHO) has provided formal guidance on policies to protect children from the harmful impact of food marketing (WHO, 2023), following the *Set of recommendations on the marketing of foods and non-alcoholic beverages to children* that were endorsed in 2010. The recommendation includes implementation of policies to restrict marketing of foods high in saturated fatty acids, trans-fatty acids, free sugars and/or salt to which children are exposed, and that such policies: be mandatory; protect children of all ages; use a government-led nutrient profile model to classify foods to be restricted from marketing; be sufficiently comprehensive to minimize the risk of migration of marketing to other media, to other spaces within the same medium or to other age groups; and restrict the power of food marketing to persuade. Restrictions on marketing are an evidence-based approach to supporting improved diets, as well as being cost-effective (World Health Organization, 2021; Boyland *et al.*, 2022b).

As of May 2022, policies on marketing restrictions toward children have been implemented in 60 countries primarily within the US and European regions (WHO, 2022). However, the majority of regulations to restrict marketing of unhealthy foods and non-alcoholic beverages to children use less effective voluntary approaches or cover limited forms of media or settings (Sing *et al.*, 2022). A recent analysis of mandatory marketing regulations in Chile and the UK, two of the few countries with mandatory restrictions, found a number of strengths but also legislative limitations, including limited coverage of both the types of marketing addressed and of children up to age of 18 (Sing *et al.*, 2022).

A key factor hampering progress on marketing regulations has been strong industry opposition and influence on policy making (Russell *et al.*, 2020; Thow *et al.*, 2020; Phulkerd *et al.*, 2022). Behind this industry influence in policy, lie perceived shared interests between key government institutions and industry related to economic objectives, which was evident in delays in introducing marketing restrictions in Chile, Australia, Fiji and Thailand (Russell *et al.*, 2020; Thow *et al.*, 2020; Phulkerd *et al.*, 2022). In Chile, industry publicly and strongly opposed marketing restrictions, in a large part by appealing to shared economic interests, (Corvalán *et al.*, 2013; Dorlach *et al.*, 2020; Mialon *et al.*, 2020) and implementation of marketing restrictions are exacerbated by limited resources, a limited government mandate, and confined policy silos within the health sector, which leads to their low capacity to engage effectively with key relevant actors within government, as observed in Malaysia, Nepal, Thailand and Fiji (Thow *et al.*, 2020; Fisher *et al.*, 2021; Ng *et al.*, 2021; Phulkerd *et al.*, 2022). Many of these factors reflect experiences in implementing nutrition policy measures more broadly (Resnick *et al.*, 2015; Cullerton *et al.*, 2016; Baker *et al.*, 2018).

As a result of these compounding factors, global progress in implementing restrictions on marketing of unhealthy food and beverages to children has been limited, and widely characterized by ‘policy stasis’: a situation in which mandatory restrictions continue to be considered, or ‘on the table’, but face consistent opposition and rarely progress to implementation. In this paper, we present a qualitative policy analysis focussed on identifying opportunities to overcome the global policy stasis on marketing restrictions. This situation can be conceptualized as a policy subsystem with two advocacy coalitions, drawing on Paul Sabatier’s ‘Advocacy Coalition Framework’ (Sabatier, 1987). Policy subsystems are defined by a policy topic and territorial scope, and are influenced directly or indirectly by a range of actors inside and outside of government who interact to produce outcomes for a given policy topic (‘advocacy coalitions’) (Sabatier, 1987; Jenkins-Smith *et al.*, 2018).

Within a policy subsystem, there is usually a dominant coalition, whose interests are broadly reflected in policy outcomes. However, where a second coalition is present, conflicting beliefs can limit the potential for policy learning within and between these coalitions—and thus hamper the potential for further policy development. Previous studies have implicitly observed the existence of two distinct coalitions with an intent to influence restrictions on marketing. One coalition tends to be oriented to encouraging regulations to restrict marketing of unhealthy foods to children, and another opposes (mandatory) regulation and often instead proposes a self-regulatory approach (Russell *et al.*, 2020; Thow *et al.*, 2020; Carvalho *et al.*, 2021; Fisher *et al.*, 2021; Ng *et al.*, 2021; Phulkerd *et al.*, 2022; Sing *et al.*, 2022). These studies indicate that actors who tend to support regulation of unhealthy food marketing are mainly situated within NGOs, academia and government departments with a mandate related to health, and usually hold beliefs related to children’s rights and the importance of supportive health environments. In contrast, the key actors that tend to support minimal regulation of marketing, or self-regulation, are often from the food industry and its representative groups, as well as government sectors with core mandates related to economic growth, including Industry, Commerce, National Planning, Agriculture and Trade. Their concerns regarding restrictions on marketing seem to reflect beliefs related to the importance of minimal regulation of industry to support economic growth. In general, studies of marketing restrictions have identified strong coalitions, which are backed by political leadership and comprised of multisectoral partnerships at all levels of government, academia, and civil society, as important facilitators of policy implementation and agenda setting (Chung *et al.*, 2022). Similar nutrition/health and economic/industry/trade coalitions have been explicitly identified in broader nutrition policy analyses informed by the Advocacy Coalition Framework (Thow *et al.*, 2018; Harris, 2019; Garton *et al.*, 2021b; Harris *et al.*, 2022).

To extend knowledge regarding marketing restrictions as a policy subsystem, the aim of this study was to further explore the nature of these (implicit) coalitions in 3 countries, and to use theoretical insights to investigate opportunities to advance policy for public health, drawing on Paul Sabatier’s hypotheses regarding policy learning (Box 1). Policy learning refers to enduring alterations in belief systems of those engaged with advocacy coalitions (Jenkins-Smith *et al.*, 2018). Sabatier posited that—in the absence of a shock to the policy subsystem, such as large shifts in socioeconomic conditions or a change in government—enabling policy learning between and within coalitions could generate policy change (Sabatier, 1987). In this situation of stasis regarding the regulation of marketing of unhealthy foods to children, in which there appear to consistently be two relatively strong and opposing coalitions and there is little evidence of shocks to the subsystem, we suggest that policy learning may provide an avenue for progress. However, little examination/empirical evidence exists in this regard.

Box 1. ***Sabatier’s hypotheses regarding moving towards change in a policy subsystem* (Sabatier, 1987)**

- *Policy-oriented learning across belief systems is most likely when there is an intermediate level of informed conflict between the coalitions. This requires: a) each have the technical resources to engage in such a debate; and that b) the conflict be between secondary aspects of one belief system and core elements of the other or, alternatively, between important secondary aspects of the two belief systems.*
- *Policy-oriented learning across belief systems is most likely when there exists a forum which is: a) prestigious enough to force professionals from different coalitions to participate; and b) dominated by professional norms.*
- *Problems for which accepted quantitative performance indicators exist are more conducive to policy-oriented learning than those in which performance indicators are generally qualitative and quite subjective.*
- *Problems involving natural systems are more conducive to policy-oriented learning than those involving purely social systems because in the former many of the critical variables are not themselves active strategists and because controlled experimentation is more feasible.*

## METHODS

This study used a qualitative policy analysis approach to examine the policy subsystem relevant to food marketing restrictions in three contexts: Thailand, Fiji and Australia. The aim of the analysis was to understand the factors and dynamics in this subsystem that give rise to policy stasis, and identify the potential to advance restrictions on marketing to children through facilitating policy learning. The study design was informed by a pragmatic research paradigm, and also influenced by an action-research method, in line with the purpose of research is to help improve policy and institutional outcomes for public health (Sadovnik, 2017).

These case study countries all represent situations of policy stasis. In Australia, there has been some regulatory progress on restricting marketing to children at the state level, but not at the national level (Ngqangashe *et al.*, 2023). In Thailand, regulation restricting marketing to children has been drafted, and is in a public hearing process (with government, civil society and private sectors) (Phulkerd *et al.*, 2022). In Fiji, detailed regulations restricting marketing to children have been drafted but not adopted (Thow *et al.*, 2020). These countries were selected for the research because they have seen some progress towards marketing restrictions—that is it is an active policy subsystem—and because this critical nutrition policy issue has been under-researched in the Asia-Pacific region. The research was led by researchers working in each country, who were familiar with the context. These three countries were also illustrative of the diversity of the region, in terms of income level, size and sub-regional context.

Data were collected via interviews with actors relevant to marketing and its health and economic implications in Australia (*n* = 24), Fiji (*n* = 10) and Thailand (*n* = 20). A semi-structured shared interview guide focussed on power and policy processes was developed by the research team, which asked about actor networks, institutional processes, power, spaces, consideration of nutrition/ health, the policy landscape and the nature of the policy issues (Appendix 1). The guide was adapted slightly for each context and translated into Thai for the fieldwork in Thailand. Potential interviewees were identified purposively by the research leads in each country and subsequently through snowball sampling. Interviewees included actors working in government (‘gov’, *n* = 22 across all countries), international organizations (‘int’, *n* = 3), NGOs (*n* = 12), academia or experts (‘experts’, *n* = 10) and the private sector (‘ind’, *n* = 7) with experience relevant to food marketing. Hour-long interviews were conducted by in-country researchers during 2020 and 2021, via zoom as required due to COVID-related measures, and were recorded and transcribed in full. Recruitment stopped once the researchers observed theoretical saturation.

The cross-country analysis presented in this paper was designed by the research team following the findings of the country-specific analyses, which identified pervasive challenges and tensions. These country-specific analyses also highlighted commonalities in experience and an unexplored opportunity to examine the potential for policy learning to provide insights for strengthening policy.

S.P. and L.Z. extracted excerpts from interview transcripts into a coding framework in excel. The codes were pre-determined, informed by the Advocacy Coalition Framework and Sabatier’s hypotheses (Box 1), and included: Beliefs, ideologies and perceptions regarding the policy ‘problem’; Characteristics of the conflict/dialogue between advocacy coalitions; actor interests; and Forums for dialogue relevant to marketing restrictions.

A.M.T. and L.Z. thematically analysed the coded data for each study country, with reference to the aims of the study. Themes were identified with reference to the study aims and framework, within and across codes and study countries. The themes were discussed iteratively with the research leads in each country (S.P., A.R. and Y.N.), and then discussed with the project team as a whole.

## RESULTS

The following over-arching themes were identified as important to understand the factors and dynamics in this subsystem that contribute to policy stasis, and identify the potential to advance restrictions on marketing to children through facilitating policy learning, and were used to structure the results: (i) policy subsystem dynamics; (ii) key characteristics of the coalitions; (iii) the nature of the dialogue between coalitions and forums in which this occurs; and (iv) implications for moving forward on marketing regulations.

### Subsystem dynamics

In all countries, health-mandated government departments had the greatest interest in regulation and marketing of unhealthy foods to children to achieve health objectives. In addition, the subsystem was characterized by a range of agencies with relevant mandates within government, and strong interests from NGOs and the food industry. However, although government health departments attempted to play a coordinating role, their mandate and capacity to effect policy change was limited.

The food standards agencies or departments in all countries, which were linked to the health sector, focused their jurisdiction on the regulation of the food itself and had a limited remit for regulating food marketing, which did not extend to restricting marketing of unhealthy foods to children. For example: *‘the food regulation system doesn’t actually regulate advertising’ AU_Gov11*

Overall the analysis indicated a regulatory lacuna; an absence of regulatory architecture within government which would address both the health and the marketing aspects of regulating marketing of unhealthy food to children. For example, in Thailand and Australia, there were existing bodies regulating advertising, but these were perceived as having limited interest and mandate to address health concerns related to advertising, resulting in minimal regulation. This dynamic also highlighted the ‘social’ rather than ‘natural’ nature of the policy subsystem. For example,

*‘Then, regarding product marketing through broadcast media, the Office of The National Broadcasting and Telecommunications Commission tended to evade the issue, calling it a social problem’ Thai_NGO4*

A major, relatively new, dynamic in regulation of marketing highlighted by interviewees in all countries was the increasingly complex and rapidly changing nature of food marketing, including increased sophistication of marketing techniques and multiplying online platforms. This was seen as stunting capacity for regulation, with governments unable to develop regulation that would be up-to-date. Interviewees from both the ‘strengthen regulation’ and the ‘minimal/self regulation’ coalitions indicated that government was struggling to identify how to effectively regulate ‘traditional’ marketing, such as television and billboards. As technology continues to evolve, it will require a multidisciplinary approach to control, which will likely be another challenge given the difficulty in accomplishing cross disciplinary action already present from a food marketing standpoint. For example:

*‘One thing we must also take into account in terms of food marketing policy is that the environment is continuously changing and the marketing mediums used by businesses are becoming more creative’ Fiji_NGO4*

Interviewees in all three countries—from both coalitions—described the food industry as very influential, and many also mentioned the advertising industry. Food industry influence was largely attributed by interviewees to effective lobbying, as well as to the economic contribution of the sector, in relation to employment and overall economic growth. For example: *‘It’s a huge sector, the economic value of the food industry is very important, as it’s sustaining life’. AU_Ind3*

There were also two key differences in the policy subsystem characteristics across countries. First, the role of ‘media’ as an industry was a notable difference across countries. In Australia, a perceived political risk arising from the impact of increased regulation on media actors was identified for government, by an interviewee in the strengthen regulation coalition: *‘government doesn’t want to have a bad relationship with the media industry… They don’t want to get them offside’ (AU_NGO3)*. In Fiji the potential advocacy role of the media in raising awareness of the draft regulation had been identified by the ‘regulate’ coalition, but interviewees noted that media had not been effectively engaged or played an active role in this way.

Second, interviewees in Thailand and Australia emphasized the importance of a change in context and government priorities with respect to public health for changes in marketing restrictions. Interviewees in both countries perceived the growing priority to address non-communicable disease through preventive health measures over the past 20 years as a shift within the policy subsystem.

### Key characteristics of the coalitions

In all countries, clear ‘strengthen regulation’ and ‘minimal/self regulation’ advocacy coalitions were evident.

The core of the *strengthen regulation coalition* were actors with an explicit health mandate or interest, including Departments of Health, health-related NGOs, and public health nutrition academics. In all countries there was a strong sense of cohesion across these groups regarding the problem that needed to be addressed through marketing restrictions—that is unhealthy diets among children and future health implications. There was also a clear articulation of the role of marketing in contributing to this problem, and the potential for restrictions on marketing to create healthier environments for children, change social norms regarding unhealthy food consumption and ultimately contribute to improved diets and health. However, there was recognition that the solution needed to be multifaceted; that marketing restrictions themselves would not ‘solve’ the problem of unhealthy diets. As a result there was less cohesion among this coalition regarding the details of the best regulatory approach.

Interviewees from all three countries, but Australia in particular, indicated that the ‘strengthen regulation’ coalition had limited appeal to political interests, compared to the food industry. For example: *‘So [politicians’] time is obviously very difficult to buy into… You’ve got to have something novel or new or interesting. We don’t have new employment figures to be able to run to them with, like the industry would be able to’. AU_TE1*. This coalition also seemed to have limited appeal to potential allies outside of the health sector. For example, there were evident shared interests related to youth and education, but the education sector seemed to be a peripheral actor due to different priorities, and in particular the lack of a mandate for health.

There was a clear shared belief among this coalition that the current approach to regulating marketing was not protecting children. This was underpinned by a belief that regulation on marketing, and improving health, were important policy priorities—often linked to frustration at the limited (self) regulation in place, which was described as ineffective.

The actors with strongest interests evident in the *minimal/self-regulation coalition* were the food industry, reported by interviewees to have lobbied strongly against government regulation on marketing on the basis of economic impacts. This included job losses and negative implications for overall economic growth, which were a shared concern with key government agencies with an economic mandate in this coalition. Other government actors outside of the health sector also had interests related to revenue from advertising (e.g. from advertising on government property, such as public transport), and sponsorship of sports or cultural events by the food industry, which aligned with government priority to encourage these. The minimal/self-regulation coalition included other actors benefiting from food industry sponsorship as a form of marketing. In particular, sporting groups in both Fiji and Australia received high profile sponsorship from industry. This created a strong disincentive for more restrictive regulation.

There were two evident shared beliefs among actors in the ‘minimal/self regulation’ coalition. The first was that marketing restrictions would hinder industry activity, which was framed as critical for achieving government (and private sector) economic objectives. As a result, marketing restrictions were possible but not preferred. For example: *‘… it is a balancing act. If we try to control advertising too strictly, then that will stifle creativity’ Thai_Gov5*. The ‘minimal/self regulation’ coalition were much more united in a key objective of maintaining the status quo, in contrast to the ‘strengthen regulation’ coalition, which interviewees indicated had diverse perspectives regarding the best regulatory approach, despite their shared clear articulation of the policy problem.

The second evident belief was that food processing is essential within the modern food system and had only a tenuous link to health outcomes. For example: *‘Most products in the supermarket undergo a form of processing and that’s primarily for food safety purposes, but it’s also for convenience and a range of other reasons’. AU_Ind4.* Industry actors effectively normalized food processing by resisting a distinction between levels of processing (e.g. between minimally processed and ultra-processed foods). They also indicated that health consequences of consumption resulted from ‘irresponsible’ decisions by consumers.

### Interface of interests between coalitions

There were three points at which the interviewees indicated overlapping interests between the two coalitions, relating to consumers, evidence and definitions. Overall, there also appeared to be a limited contest of core beliefs between the coalitions.

A key actor group that appeared to sit between coalitions in all countries was consumers. This included formal consumer representative agencies, which had shared interests with the ‘strengthen regulation’ coalition relating to health, access to information, and access to affordable food (rather than specifically healthy food). It was apparent that consumers were seen by interviewees from different coalitions in diverse ways. On the one hand, they were positioned as a powerful (potential) actor for both coalitions to win over. For example:

*‘I think communities are influential… because from a government perspective, they have to meet public expectations because they’re voted in. From an industry perspective, you have to meet what the population wants…’ AU_Ind1*

Further to this, successes in improving regulation on marketing was sometimes attributed to consumers being supportive of the goals of that coalition.

On the other hand, consumers were also positioned as the problem, or the victims of marketing—effectively as a challenge for each coalition to overcome. On the ‘strengthen regulation’ side, this took the form of consumers not appreciating the need for healthier environments. On the ‘minimal/self regulation’ side, this took the form of there being no need for marketing restrictions, if consumers were more aware and demanded healthier food. Industry consistently pointed to market demand for (unhealthy) food as a critical influence on their product range, and emphasized the role of consumers in driving change. For example: *‘if the consumer demands a product with less sugar, industry will certainly respond to meet that demand’ (Thai_Ind1).*

There was a second point of interface between the coalitions regarding evidence. On the ‘strengthen regulation’ coalition side, there were frequent comments regarding the challenge of generating evidence that would be compelling in the face of industry opposition. This included the limited evidence available for the impact of marketing on diet and health outcomes, which were described as ‘distal’ and ‘impossible’. Interviewees also highlighted the importance of evidence regarding economic impact for gaining support for regulations—in particular, the revenue implications as well as long term health care savings and productivity gains. On the ‘minimal/self regulation’ coalition side, industry acknowledged the problem of poor diets and health, but consistently cast doubt on whether marketing restrictions would really achieve the goal.

Third, a key feature of the dialogue between the coalitions in all three countries was a focus on the need to more clearly define ‘unhealthy food’. Several interviewees noted that definitions were an unresolved issue, and became a sticking point for negotiations on regulatory approach. The ‘minimal/self regulation’ coalition repeatedly pointed to the inability of government to consistently define healthy and unhealthy foods as an indication that the government lacked capacity to effectively regulate marketing. The strengthen regulation coalition did not have the same issue and for the most part, could clearly delineate between healthy and unhealthy foods. They also did not emphasize a need to divide groups into healthy and unhealthy to effectively regulate the marketing, in contrast to industry. For example:*‘…we spend a lot of time arguing about the definition of which foods are healthy and unhealthy… Actually it wouldn’t matter where you draw the line… reducing some of the unhealthy food advertising, is what we’re trying to achieve here’. (AU_Gov7).*

Overall, rather than a contest of core beliefs, there seemed to be a common belief within both coalitions that, in general government intervention in the market is appropriate. The issue of difference was whether restricting marketing would be worth it. In other words: industry would be negatively impacted by restrictions on marketing, but would this be outweighed by the benefit to health and children? And, was it the governments’ role to pro-actively support consumers to improve their diets? This tension regarding trade-offs was constantly being navigated and negotiated, but overall tended to fall on the side of the dominant ‘minimal/self regulation’ coalition. For example:

*‘For [industry], their argument is always that more marketing restrictions and regulations in general does not favor the industry, which means they will lose out on profit… they use that to influence the Ministry of Trade and the Ministry of Health as well… food industries contribute to the GDP’. FJ_NGO1*

This argument regarding trade-offs was further buttressed by strong industry message that self-regulation is a very effective approach to achieve the policy objectives. In Australia, in particular, interviewees in the ‘strengthen regulation’ coalition indicated that industry had successfully convinced government that the self-regulatory approach is effective.

### The spaces and structures in which dialogue between coalitions occurs

The dialogue and engagement between the coalitions occurred in both informal and formal, as well as open and closed, spaces. The primary space in which engagement between coalitions took place appeared to be government-mediated consultative spaces. For example, meetings regarding the current (self) regulatory approach to marketing broadly, often convened by (government) food standards bodies, and consultations on proposed restrictions. Interviewees also referred to consultations convened by non-government actors within the ‘strengthen regulation’ coalition, with a view to introducing policy change.

Industry organizations and food standards agencies both seemed to form a bridge between industry and government in all countries, namely through creating formal and informal spaces for dialogue that were perceived as more neutral than either industry-led or health-led spaces. The overall mandate for these institutions differed; the food standards agency was mandated to convene consultations (dialogues) on policy proposals, whereas the industry organizations were mandated to lobby (initiate dialogues with) government representing industry interests, through both formal and informal channels. However, interviewees in all three countries indicated that government had also collaborated with an industry organization to convene a dialogue, which was successful in bringing a wider range of actors to the table. Similarly, interviewees reported that consultations convened by food standards agencies had strong industry representation, and a space in which government actors had established relationships with industry actors.

The formal spaces for dialogue were seen by public health interviewees in all three countries as being undermined by the direct access that industry actors had to decision makers within their coalition, who were often influential. Public health interviewees attributed this access to both the economic contribution of industry and the much greater resources, compared to public health actors. In contrast, Industry interviewees in Fiji and Australia talked about direct informal discussions with decision makers as a normal part of their engagement on issues of relevance. For example: *‘I think if the consultations don’t quite progress properly… the industry then has to go to the Ministry of Industry and Trade for help. That’s what’s happened in the past because the Ministry of Health was just not receptive to the industry’s views’. (Fiji_Ind1)*.

### Implications for moving forward on marketing regulations

The progress in regulations to restrict marketing that interviewees described in this study had four key features relevant to cross-coalition engagement, which could inform future learnings in the study countries as well as other jurisdictions seeking to progress marketing restrictions. These related to three of Sabatier’s hypotheses regarding moving towards change within this more ‘social’ policy subsystem: changing the goal to enable informed conflict through embracing incremental policy change; shifting performance indicators through taking a ‘positive’ approach to defining foods; shifting performance indicators through new approaches to monitoring; and changing the nature of the forum through bringing in new expertise that would help to support more constructive dialogue.

First, interviewees in Thailand and Australia had observed progress through incremental policy change that was focussed on specific sub-areas of policy related to marketing restrictions (Table 1). In particular, this approach appeared to reduce the scale of opposition and the negative trade-offs for industry were more easily managed. For example, in Thailand, focussing on school food environments was reported to have been easier to implement than a broad-scale approach (despite challenges with enforcement). In Australia, interviewees had observed success through a focus on restricting advertising on government assets, such as public transport, and restricting advertising associated with sporting events.

**Table 1:** Examples of interviewee statements indicating opportunities for policy learning

	Australia	Fiji	Thailand
Changing the goal to enable informed conflict: Incremental policy change	*‘you can progress in smaller steps towards that outcome… Starting where there is some will to make those restrictions, and government assets is one that’s worked for a number of jurisdictions’. AU_Gov6*	*‘If [NGOs] could be involved from the very beginning, then we can create more awareness on food marketing policies and its provisions and the implications this will have on our consumers’. Fiji_NGO4*	
Shifting performance indicators: Taking a ‘positive’ approach to defining foods	*‘when we were framing up the policy… Our minister [wanted] to be very focussed on the positive, so healthy eating, as opposed to unhealthy. And so… instead it was here are the things you can advertise and everything else, you can’t’. AU_Gov6*	*‘we really need to consider repositioning ourselves and focussing on research that asks questions like ‘how can we reposition the healthy, nutritious food from the Pacific?’ ‘how can we market it better and promote the local healthy foods against the imported processed ones?’ I think this is an innovative way of marketing healthier food options and we have not really capitalized on doing this in the region’. Fiji_INT2*	*‘It is hard for [industry] to accept that their products that are currently on the market are harmful to the population’s health in any way. … By using the 4-way classification based on nutrient profile, it gives the food industry a pathway to our goal of healthier eating. We shouldn’t portray it as an either/or proposition. It should be a gradient toward full compliance’. Thai_Gov3*
Shifting performance indicators: New approaches to monitoring	*‘The politicians do believe that if the industry says they’re doing a good job, then they’re doing a good job’ AU_NGO2* *‘so you remove the ads in the London Tube, and then the measurements there need to include things that are of importance to the government. So, loss of revenue, for example… the impacts [must] look beyond health, they also look at the economic impact’. AU_Gov7*	*‘the Ministry of Education [should] undertake enforcement and monitoring of these policies – or at least they should drive this and work closely with the Ministry of Health to enforce and monitor Fiji’_NGO4*	*‘Before, there were censors to monitor the broadcasts… That was when the Public Relations Department had real clout. …But that self-monitoring was onerous and arbitrary, so they switched to the rating system. But the rating system hasn’t really worked…’ Thai_NGO4*
Changing the nature of the forum: Need for new expertise	*‘[government have] got very little capacity, very few people who have any content expertise in this area’ AU_TE3*	*‘Industry is often willing to make the change, but they need to be consulted and we need to understand how they work and what works for them, etc’. Fiji_Gov1* *‘At the moment, doctors are also serving as technical experts, but we don’t need doctors in our area. We need policy people, social researchers… Food is a social issue. When you’re talking about policy implementation, we need a social sector. The people who deal with the food, not the people who deal with the disease’. Fiji_Gov1*	*‘There was some suggestion that we needed to consult more with the Children and Youth councils and the Committee on Protection of the Rights of the Child’ Thai_Gov3* *‘Whoever is going to champion the issue of food marketing needs to have knowledge and expertise, and thoroughly understand marketing strategies’. Thai_NGO4*

Second, taking a ‘positive’ approach to designating the foods that would be subject to marketing restrictions could circumvent debates on the definitions of unhealthy food. Interviewees reported that reorienting the discussion from what *couldn’t* be advertised to what *could* be advertised—with a focus on fresh and whole foods—could create a shared focus on healthy foods (Table 1). Similarly, interviewees in Thailand from the strengthen marketing coalition observed that using a graded or staged approach to scale up the definition of unhealthy food had gradually created a more positive engagement with industry. Interviewees perceived this approach as creating a common platform for dialogue, given the very different starting points of the two coalitions.

Third, it was evident in all three countries that interviewees saw potential for monitoring and data to create a credible threat of regulatory escalation (Table 1). In all the study countries, interviewees—particularly those from NGOs—identified the limited monitoring of the existing situation as a contributor to policy stasis. There was a clear divide in beliefs about the efficacy of self-regulatory approaches between the coalitions, and those in the ‘strengthen regulation’ coalition saw monitoring as an avenue to create an evidence-based dialogue on what the impact of the current approaches are, with a view to policy change. Comprehensive monitoring was also perceived as enabling a holistic assessment of trade-offs involved in the regulation of marketing, which would ensure that the beliefs of the different coalitions were recognized. In particular, assessing not only the impacts on children’s viewing, diets etc, but also economic implications, such as impacts on revenue and industry, would provide evidence not only for health impact but also impacts on government revenue and industry actors.

Fourth, the interviewees identified a need for new expertise in the strengthen regulation coalition, including deeper engagement with new media and emerging marketing approaches. This was linked to a recognition that public health expertise was lacking an understanding of both the dynamic nature of the evolving marketing landscape, and also the existing regulatory environment. Related to this, several interviewees aligned with the ‘strengthen regulation’ coalition identified a need for engagement and dialogue to include a more diverse range of actors, in order to generate change, particularly those with an interest in children’s rights.

## DISCUSSION

This study identified two advocacy coalitions regarding the regulation of marketing of unhealthy food to children in Fiji, Australia and Thailand that were similar both to each other, and to those implicitly described elsewhere (Carvalho *et al.*, 2021; Fisher *et al.*, 2021; Ng *et al.*, 2021). The dialogue between the coalitions centred on trade-offs between the public health and economic objectives of government, similar to the strong economic discourse that has been identified as hampering progress in previous analyses (Russell *et al.*, 2020; Fisher *et al.*, 2021). The consistent policy stasis that was evident in these case study countries in part reflected a regulatory lacuna with respect to marketing and a separation of mandates between health and various regulatory agencies with a remit related to (but not specifically for) regulation of food marketing to children. This has also been highlighted in Brazil, where the lack of an agency with a clear mandate and competency for regulating marketing hampered progress (Carvalho *et al.*, 2021).

By engaging with the policy learning hypotheses related to the Advocacy Coalition Framework, we were able to identify potential avenues to move beyond the prevailing policy stasis and support restrictions on marketing of unhealthy food to children. These included taking an incremental approach to policy change, focusing on permitted (rather than restricted) foods and investing in new public health expertise related to emerging marketing approaches. The analysis also identified the potential contribution of scaling up monitoring of impacts to support policy learning and change. This resonates with findings from a study in Malaysia, where evidence regarding the lack of impact of self-regulation generated support for a regulatory approach to marketing restrictions (Ng *et al.*, 2021). More broadly, limited allocation of resources and capacity for effective monitoring has been identified as a major limitation of existing policies for restricting marketing (Olstad *et al.*, 2020; Sing *et al.*, 2022). Other theoretical perspectives can also provide insights on factors that may help to overcome policy stasis. For example, the multiple streams approach highlights the role policy entrepreneurs can play in increasing participation of diverse actors in dialogues, and negotiating on policy design (Kingdon, 1984).

Although the coalition dynamics described in this paper provide insights for navigating the policy process surrounding marketing restrictions, it is important to note that there are likely to be underlying networks at play that may undermine or limit attempts at developing policy learning between coalitions. Some of the policy dynamics described also reflect corporate political activity, including lobbying, establishment of relationships, policy substitution and use of information by industry (Mialon *et al.*, 2020; Gilmore *et al.*, 2023). For example, in Fiji, previous research identified food industry activity to create a favourable regulatory environment, particularly through establishment of relationships with the community, the media and with policy makers; and proposing alternative policy approaches (Mialon *et al.*, 2016). An analysis of marketing restrictions in Brazil identified the importance of social networks and historical relationships (including university and professional connections) that could be leveraged for political influence (Carvalho *et al.*, 2021). Consistent with Sabatier’s framework, policy networks are likely to be formed among actors with shared beliefs and ideologies, and embedded in more complex relationships than those relevant to a single policy issue—even as they reinforce existing network cohesion related to a specific policy process (Henry, 2011). The strong economic orientation of the ‘minimal/self regulation’ coalition suggests that for a policy learning approach to effectively move marketing restrictions forward, public health advocates need to engage more strategically with the economic policy and politico-economic context through both formal institutional mechanisms such as impact assessments and also informal relationships (Garton *et al.*, 2021a). A recent analysis in Canada that included (unsuccessful) restrictions on marketing highlighted the importance of cultivating greater acceptability of beliefs related to societal responsibility as a means to foster policy change for NCD prevention (Nykiforuk *et al.*, 2019). Although fostering such a change in beliefs and ideas is challenging, insights from institutional approaches to political economy highlight the potential for changes in ideas to occur through attention to framing and creation of spaces in which there can be explicit acknowledgement of conflicting ideas (Campbell, 1998). One of the specific contributors to policy learning identified by Sabatier was the relative prestige of the spaces for dialogues, which wasn’t raised explicitly in the interviews. However, the findings regarding broad industry representative organizations and food standards organizations as ‘bridging’ the two coalitions may implicitly reflect perceptions of representation or relatively neutrality that enable constructive dialogue and conflict. This issue of representation was addressed in Chile with the creation of citizen forums by the Ministry of Health in conjunction with NGOs, as a space for inclusive dialogue (Villalobos Dintrans *et al.*, 2020).

This comparative policy analysis drew on the Advocacy Coalition Framework to conduct an action-oriented study based on interview data with participants across a range of sectors. By examining three diverse contexts facing similar challenges in restricting marketing of unhealthy food to children this study has highlighted several specific approaches to overcome policy stasis. The main limitation of this study was our inability to ascertain different types of actor beliefs in detail due to the secondary analysis that was conducted; the study tools weren’t designed with this analysis in mind. In addition, the study drew only on interview data, which were not triangulated with documentary or quantitative data (such as statistics on the economic contribution of the industry). The case studies were also limited to three countries that had experienced partial success, and did not include a country with mandatory marketing restrictions. However, given the high percentage of countries globally with non-mandatory regulation, and even higher number of countries that have not successfully adopted any marketing regulation, learning from countries with partial progress is likely to provide relevant learnings to countries that have not yet experienced any policy progress in this area.

## CONCLUSION

This analysis of the advocacy coalitions related to restricting marketing of unhealthy foods to children in Australia, Thailand and Fiji has identified beliefs and ongoing approaches to dialogue that have contributed to policy stasis on this issue. Policy learning that enables policy progress on restrictions on marketing of unhealthy foods to children could be supported by taking an incremental approach to policy change, focusing on permitted (rather than restricted) foods, investing in new public health expertise related to emerging marketing approaches and scaling up of monitoring of impacts.

## Supplementary Material

daad136_suppl_Supplementary_Appendixs_1

## Data Availability

The datasets generated and/or analyzed during the current study are not publicly available to ensure privacy of interviewees. Access to anonymized interview transcripts and archival data is available from the corresponding author on reasonable request.
