# Supplementary material for: Protecting children from unhealthy food marketing: a comparative policy analysis in Australia, Fiji and Thailand
Source: Health Promot Int. 2023 Nov 27;38(6):daad136. doi: 10.1093/heapro/daad136 (PMC10681351; doi:10.1093/heapro/daad136)
Supplement: daad136_suppl_Supplementary_Appendixs_1 [file daad136_suppl_supplementary_appendixs_1.docx]

Appendix 1: Interview guide (generic version, slightly adapted for all countries and translated for Thailand)

**Interview guide**

1. Could you start by telling me about your involvement in food marketing policy?

Prompts: research, advocacy, lobbying, influencing policy

- 1. Are you involved as an individual or as part of an organization?
  2. If part of an organization, what is your specific role in the organisation with regard to food marketing policy?
  3. Are there roles you think you should be playing with respect to the making of policies on food marketing but aren’t? Which ones? Why?
  4. Are these roles played by anyone else at the moment? Who?
  5. What needs to happen to enable you to play these roles?

1. What are your priorities with regard to food marketing policy? Why are these important?
   1. What kind of resources do you have to help you meet these goals / priorities?

Prompts: technical expertise, research, access to policy makers;

1. Who do you think is influential in shaping food marketing policy in [country]?

Prompts: Food industry, Advertising industry, Department of Health, Department of Communications, Department of Education, Statutory bodies

- 1. What makes them influential?

Prompt: economic resources, legitimacy/expertise, direct access, social networks, personal relationships, status/prestige (symbolic)

- 1. Can you give me an example of the aspect of food marketing policy you think they can influence?

Prompt: types of foods that are/are not regulated, the design of the regulation etc.)

- 1. What structures/mechanisms do they use to influence?

Prompt: direct lobbying, one-on-one meetings; provide evidence

- 1. In what spaces does the influencing of decision making on food marketing policy take place?

Prompts: public spaces, closed spaces, committees, private meetings

- 1. Have you experienced or observed any barriers in accessing these Spaces?

1. Other than the people we have discussed so far, do you work with any other people in food marketing policy – e.g. government departments, industry organizations, technical experts or non-government organisations?
   1. What drives these collaborations?

Prompts: similar goals; ideas

- 1. Where do these collaborations take place?

Prompts: Are they public/private spaces? Are they by invitation only?

- 1. Are there any people you would like to work with but haven’t been able to? Who and why?

1. In your opinion, do you think health is considered when food marketing policy decisions are made in [country]? How?
   1. If food marketing policy were to support better health outcomes, what do you think should be the focus of the policy? Why are these important?

Prompts: nutrient profiles, types of foods that are regulated, types of regulation, target groups, platforms

- 1. What kind of resources are needed to facilitate this?

Prompts: technical expertise, research, access to policy makers;

- 1. Do you think that focusing attention on specific food categories such as “ultra-processed foods” would help in orientating food marketing policy towards health outcomes? How?
  2. Do you engage or collaborate with decision makers or (influential people) to keep health/nutrition on the food marketing policy agenda? If yes, in what way?

Prompt: participating in expert panels/committees; comment on draft policy

- 1. Do your goals align with these influential actors? If not, are they responsive to engaging with your objectives? Do you have any tools for advancing your goals?

1. Food marketing practices are always evolving (e.g. online marketing, gaming)
   1. What are the policy challenges?
   2. Are you aware of discussions about adapting food marketing policies to this new context of food marketing? Or any general changes? What does the process look like?
   3. Who would likely support or oppose these changes?
   4. What could be your role in this?
2. International best practice for health recommends policies that restrict food marketing to children. Do you think the current food marketing policies in [country] are in line with this?
   1. What are the challenges or gaps?
3. Is there anything else you would like to tell me about food marketing policy that I haven’t asked about?
4. Is there anyone else you think I should be speaking with?
